# CADASIL: Ultrastructural insights into the morphology of granular osmiophilic material

**DOI:** 10.1002/brb3.624

**Published:** 2017-02-22

**Authors:** Teresa Lorenzi, Michele Ragno, Francesca Paolinelli, Clara Castellucci, Marina Scarpelli, Manrico Morroni

**Affiliations:** ^1^ Section of Neuroscience and Cell BiologyDepartment of Experimental and Clinical MedicineSchool of MedicineUniversità Politecnica delle MarcheAnconaItaly; ^2^ Division of NeurologyMadonna del Soccorso HospitalSan Benedetto del TrontoItaly; ^3^ Division of PathologyDepartment of Clinical and Molecular SciencesSchool of MedicineUniversità Politecnica delle MarcheAnconaItaly; ^4^ Section of Pathological AnatomyDepartment of Biomedical Sciences and Public HealthSchool of MedicineUniversità Politecnica delle MarcheUnited HospitalsAnconaItaly; ^5^ Electron Microscopy UnitUnited HospitalsAnconaItaly

**Keywords:** CADASIL, electron‐lucent halo, granular osmiophilic material deposits, mixed GOM, transmission electron microscopy

## Abstract

**Introduction:**

Cerebral autosomal dominant arteriopathy with subcortical infarcts and leukoencephalopathy (CADASIL) is a hereditary systemic vascular disorder. Granular osmiophilic material (GOM) is its ultrastructural marker. We reviewed tissue biopsies from CADASIL patients to establish whether ultrastructural observations help clarify the pathogenic mechanism of CADASIL. Given the resemblance of the GOM deposits to the immunoglobulin deposits seen in glomerulonephritis and focal segmental glomerulosclerosis (FSGS), their morphologies were investigated and compared.

**Methods:**

Skin, skeletal muscle, kidney, and pericardium tissue biopsies from 13 patients with a clinical and molecular diagnosis of CADASIL, and kidney biopsies from five patients with IgA nephropathy and five patients with primary FSGS were subjected to ultrastructural examination.

**Results:**

In CADASIL patients, several GOM deposits from all sites were partially or totally surrounded by an electron‐lucent halo. The deposits frequently had a more electron‐dense portion with a regular outline on the inner side and a less osmiophilic, looser outer side displaying a less regular profile. The uniformly dense deposits tended to be more osmiophilic if located close to the cell membrane and less osmiophilic if laid farther away from it. The immunoglobulin deposits from the glomerulonephritis and FSGS patients lacked both the granular pattern and the halo.

**Conclusions:**

This study demonstrates that GOM deposits may have a nonuniform morphology and describes in detail an electron‐lucent halo surrounding several of them. It is conceivable that the halo is the morphological evidence and possibly the cause of an aberrant NOTCH3 processing, already suspected to be involved in CADASIL.

## 1 Introduction

Cerebral autosomal dominant arteriopathy with subcortical infarcts and leukoencephalopathy (CADASIL) is a hereditary systemic vascular disorder involving mainly small and middle‐sized arteries of the microcirculation. Although all organs are affected, the symptoms are almost exclusively neurological. The main clinical manifestations of the disorder include recurrent transient ischemic attacks (TIAs), stroke, psychiatric disturbances, cognitive defects, epilepsy, and migraine, which lead to dementia and premature death.

CADASIL is caused by mutations in the *NOTCH3* gene, which is located on chromosome 19 (Tournier‐Lasserve et al., 1993). The *NOTCH3* gene encodes for the transmembrane receptor NOTCH3 (neurogenic locus notch homolog protein 3) that is expressed almost exclusively in vascular smooth muscle cells (VSMCs) and pericytes (Joutel et al., 1996). The disease induces progressive degeneration and eventual disappearance of VSMCs and accumulation of the extracellular domain of NOTCH3 protein (N3ECD) in the walls of affected arteries (Brulin, Godfraind, Leteurtre, & Ruchoux, 2002; Joutel, 2011; Joutel et al., 2000). A distinctive early feature of CADASIL is the buildup of deposits of granular osmiophilic material (GOM) on or close to degenerating VSMCs (Ruchoux et al., 1995). N3ECD is a component of GOM (Ishiko et al., 2006; Yamamoto et al., 2013). CADASIL mutations have been reported to enhance N3ECD multimerization (Opherk et al., 2009), suggesting that they are neomorphic, that is, they confer a novel function on the mutant receptor. It has also been shown that the pathophysiology of the disorder is associated with hypomorphic NOTCH3 activity in VSMCs, consistent with loss of NOTCH3 function (Arboleda‐Velasquez et al., 2011; Moccia et al., 2015). However, it is still debated if GOM deposits are involved in CADASIL pathogenesis or only represent an epiphenomenon in this disease (Erro et al., 2015; Moccia et al., 2015).

For decades, electron microscopy (EM) has been the only technique capable of identifying biological particles in cells and to provide tracking information. Despite key advances in biochemical and molecular analytical methods, EM remains the most informative morphological approach to the dynamic aspects of several catabolic processes. EM detection of double membrane‐enclosed vesicles (autophagosomes) containing cytoplasmic material and of lysosomes fused with them—having the function of degrading the engulfed cellular components—has led to the definition of autophagy (Eskelinen, Reggiori, Baba, Kovács, & Seglen, 2011). Autophagy prevents accumulation of unwanted elements, such as aberrant protein aggregates, by selective elimination (Klionsky & Emr, 2000). Since impaired/disrupted autophagy has been linked to several human disorders (Choi, Ryter, & Levine, 2013), EM has been providing crucial data to understand a variety of diseases (Nixon et al., 2005; Perrotta, 2013). Studies of extracellular protein aggregates similar to those seen in CADASIL have been performed by EM in amyloidosis diseases (Tosoni, Barbiano di Belgiojoso, & Nebuloni, 2011) such as Alzheimer's (Yamaguchi et al., 2000) and Parkinson's (Baba et al., 1998). In these cases, too, EM has provided important etiological insights.

GOM has already been investigated by EM (Baudrimont, Dubas, Joutel, Tournier‐Lasserve, & Bousser, 1993; Kalimo, Ruchoux, Viitanen, & Kalaria, 2002; Lewandowska, Dziewulska, Parys, & Pasennik, 2011; Morroni & Lorenzi, 2013; Ragno, Nardi, Manca, Morroni, & Trojano, 2013; Ragno et al., 2012, 2013; Ruchoux et al., 1995; Schröder, Züchner, Dichgans, & Nagy, 2005). The aim of this study was to gain further insights into CADASIL pathobiology by in‐depth analysis of the ultrastructure of GOM deposits. The examination of biopsy samples from 13 patients with the clinical, genetic, and ultrastructural features of CADASIL confirmed earlier reports of an electron‐lucent halo around several GOM deposits and their nonuniform osmiophilia (Ishiko, Shimizu, Nagata, Ohta, & Tanaka, 2005; Lewandowska et al., 2011; Ruchoux et al., 1995).

The observation of GOM in a renal glomerulus from a CADASIL patient with renal symptoms (Ragno et al., 2012) and its resemblance to the mesangial immunoglobulin deposits found in glomerulonephritis and focal segmental glomerulosclerosis (FSGS) (LaPoint, Patel, & Rubio, 2000) prompted us to compare their respective ultrastructures.

## 2 Material and methods

### 2.1 Standard protocol approvals, registrations, and patient consents

This study was approved by the Ethics Committee of Università Politecnica delle Marche and conducted in accordance with the Helsinki Declaration of 1975, as revised in 2008.

Written informed consent was obtained from all participants and is recorded on file. Patients were informed that the biopsies were collected for diagnostic and research purposes and that the results would be anonymized.

### 2.2 Patients

Biopsy samples from 13 subjects (eight men and five women, age range 45–64 years, mean 53 years) with the clinical, genetic (all mutations altering the number of cysteine residues in the N3ECD of NOTCH3), and ultrastructural characteristics of CADASIL disease were reviewed. Patient age and sex, sample type, and molecular findings are reported in Table 1.

**Table 1:** Patients, samples, and genetic findings from 13 CADASIL patients (from Morroni et al., 2013)

Pt No.	Sex	Age/years	Sample	GOM	Mutation in	Amino acid change
1^a^	M	51	Skeletal muscle	+	Exon 3	C108S
2^a^	M	57	Skin	+	Exon 3	C108S
3^a^	F	50	Skeletal muscle	+	Exon 3	C108S
4	M	45	Skin and kidney	+	Exon 19	R1006C
5	F	64	Skin	+	Exon 2	R54C
6	M	47	Skin	+	Exon 10	G528C
7	M	53	Skin	+	Exon 10	G528C
8	M	48	Skeletal muscle	+	Exon 10	G528C
9	F	46	Skin	+	Exon 3	R110C
10	F	56	Pericardium	+	Exon 10	G528C
11	M	59	Skin	+	Exon 4	R141C
12	F	54	Skin	+	Exon 10	G528C
13	M	54	Skin	+	Exon 6	R332C

Pt: patient.

+ presence of GOM.

a Patients from the same family (siblings).

A punch skin (*n *= 8) or a muscle biopsy (*n *= 3) was available for 11 patients; a pericardial tissue fragment obtained during open heart surgery for valve replacement was available for another (#10; Table 1); and a skin and a kidney biopsy (the latter obtained at a later time to investigate renal symptoms) were available for a further patient (#4; Table 1). Muscle (#1 and #3) or skin (#2) tissue were examined in three affected siblings (Table 1). These data have recently been published (Morroni et al., 2013; Ragno et al., 2012).

Since the GOM found in a renal glomerulus from a CADASIL patient with renal symptoms (Ragno et al., 2012) resembled the mesangial immunoglobulin deposits found in glomerulonephritis and FSGS patients (LaPoint et al., 2000), their respective morphologies were examined and compared. To do so, kidney biopsies from 10 patients with IgA nephropathy and primary FSGS (five each; six men and four women, age range: 35–61 years, mean: 40 years), the renal pathologies that are more frequently associated with such types of deposits, were analyzed.

Immunofluorescence microscopy confirmed mesangial immunostaining for IgA and IgM in the two sets of patients, respectively.

### 2.3 Immunofluorescence and transmission electron microscopy (TEM)

Renal tissue routine immunofluorescence was performed on 4‐μm‐thick cryostat sections using polyclonal FITC‐conjugated antibodies to IgA, IgM, and C3 (Dako Corp., Carpinteria, CA, USA).

Tissues for TEM observation were fixed in 2% glutaraldehyde/2% paraformaldehyde in 0.1 M phosphate buffer, postfixed in buffered osmium tetroxide, dehydrated in graded alcohols, and embedded in an Epon‐Araldite mixture. Semithin sections (2 μm) were obtained with a MICROM HM 355 microtome (ZEISS, Oberkochen, Germany) and stained with toluidine blue to select arteries of appropriate size for thin sectioning. Thin sections were cut with an MTX ultramicrotome (RMC, Tucson, AZ, USA), stained with lead citrate and examined with a CM10 transmission electron microscope (Philips, Eindhoven, The Netherlands).

## 3 Results

**Figure 1:**
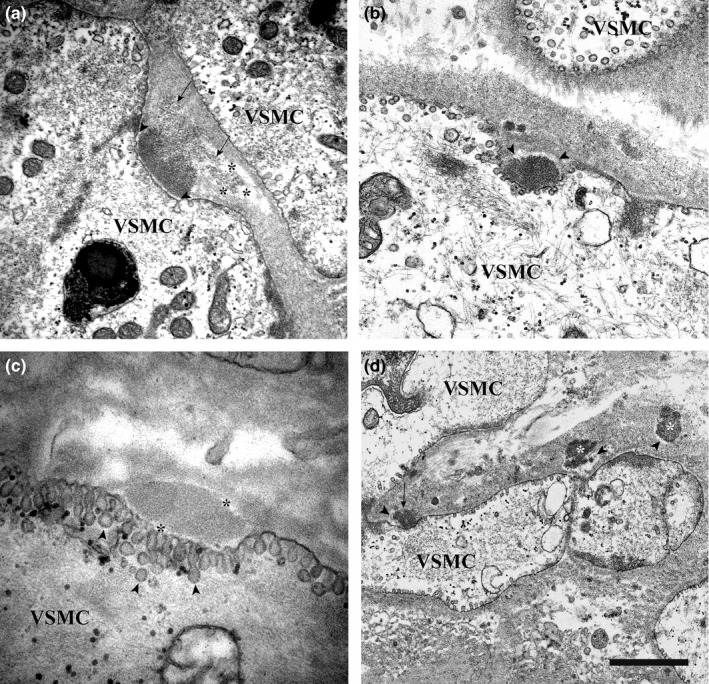
Transmission electron microscopic images of skin and renal biopsies from GOM‐positive patients. (a) Skin biopsy (patient #2). GOM in an infolding of a VSMC is separated from the cell membrane by a thin electron‐lucent halo (arrowheads). Note, the inhomogeneous density of the GOM: the inner side is denser and has a smooth profile, the interstitial side (arrows) is less osmiophilic, looser, and shows an irregular profile. The right portion of the less osmiophilic area is surrounded by an electron‐lucent halo (asterisks). (b) Renal biopsy (patient #4). GOM in an infolding of a VSMC. An electron‐lucent halo (arrowheads) separates it from the interstitium (from Figure 3 of Ragno et al., 2012) (permission granted by the Publisher). (c) Skin biopsy (patient #12). GOM in an infolding of a VSMC. The deposit is completely surrounded by an electron‐lucent halo (asterisks). Numerous pinocytotic vesicles (arrowheads) are clearly evident in proximity to the GOM deposit. (d) Renal biopsy (patient #4). Three GOM deposits: one is in close contact with a VSMC (arrow), the other two are free in the interstitium (asterisks). All three deposits are partially surrounded by an electron‐lucent halo (arrowheads). Scale bar: a, 840 nm; b, 600 nm; c, 370 nm; d, 1.6 μm.

**Figure 2:**
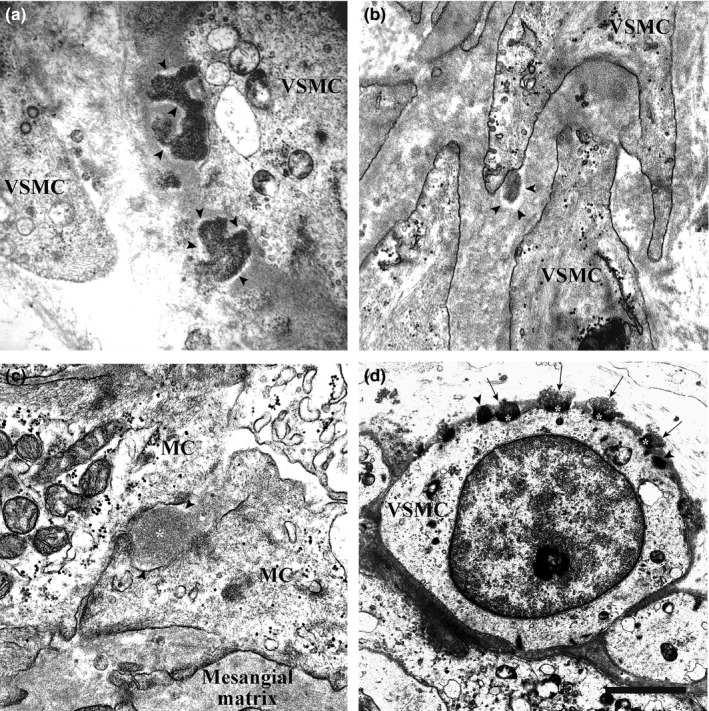
Transmission electron microscopic images of skeletal muscle, pericardial, and renal biopsies from GOM‐positive patients. (a) Skeletal muscle biopsy (patient #3). Two GOM deposits with very irregular profiles close to a VSMC are partially surrounded by an electron‐lucent halo (arrowheads). (b) Pericardial biopsy (patient #10). A small GOM deposit surrounded by an electron‐lucent halo (arrowheads) in an infolding of a VSMC process. (c) Renal biopsy (patient #4). GOM (asterisk) between two mesangial cells (MC) of a glomerulus from a CADASIL patient with renal symptoms. Two lighter areas around the GOM deposit are evident. (d) Skeletal muscle biopsy (patient #3). Numerous GOM deposits located on the cell membrane of a VSMC; most have an inhomogeneous morphology: the site facing the cell membrane is denser and has a smoother profile (asterisks), the site facing the extracellular matrix is less dense and has an irregular profile (arrows). Two GOM deposits show homogeneous density (arrowheads). Scale bar: a, 900 nm; b, 860 nm; c, 620 nm; d, 2.1 μm.

The GOM deposits identified in the 13 CADASIL patients all had the ultrastructural features described in the literature (Baudrimont et al., 1993; Kalimo et al., 2002; Lewandowska et al., 2011; Morroni & Lorenzi, 2013; Ragno et al., 2012, 2013, 2013; Ruchoux et al., 1995; Schröder et al., 2005). They were variable in size (from 0.2 to 0.8 μm), shape, and number; they were made up of granules 10–15 nm in diameter; and were found at extracellular sites, most often near VSMC indentations or close to VSMCs of small and medium‐size arteries. Occasionally, they were detected in capillaries—where they were predominantly located very close to pericytes—and vein walls. Several pinocytotic vesicles were seen close to the deposits in the indented cytoplasmic membrane of smooth muscle cells and pericytes. Interestingly, the deposits found in VSMC infoldings were often separated from the cell membrane by an electron‐lucent halo 20–90 nm in thickness (Figure 1a). In some cases, the halo was only on the outer side of the deposit (Figure 1b), whereas at other times it surrounded it completely (Figure 1c). GOM deposits located in the interstitium also displayed the halo (Figure 1d). The halo closely followed the deposit contour also in cases where it was irregular (Figure 2a). Deposits partially or completely surrounded by a halo were found in all tissue samples (Figure 2b). In the kidney biopsy (patient #4), GOM was detected in the arteries (Figure 1b,d) as well as in the glomerulus, had a granular pattern and was surrounded by an electron‐lucent halo that separated it from mesangial cells and the mesangial matrix (Figure 2c).

**Figure 3:**
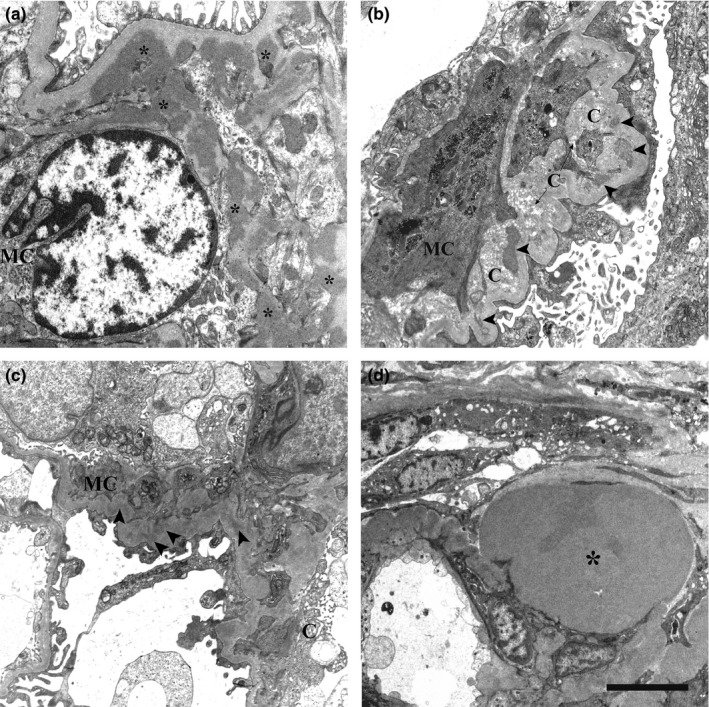
Transmission electron microscopic images of renal biopsies. (a) IgA nephropathy. Several electron‐dense mesangial deposits (asterisks) are shown. They lack the electron‐lucent halo. (b) Chronic IgA nephropathy. Mesangial deposits with varying degrees of resorption (arrowheads) close to collagen fibers are evident. Around them, the electron‐lucent halo is not detectable. (c) FSGS. Small electron‐dense IgM deposits in the mesangium are not surrounded by the electron‐lucent halo (arrowheads). (d) FSGS. A large electron‐dense hyaline material (asterisk) in paramesangial position is shown. The electron‐lucent halo is not visible. MC, mesangial cell. C, collagen fibers. Scale bar: a, 2 μm; b, 750 nm; c, 2.5 μm; d, 1 μm.

The immunoglobulin deposits in the biopsy samples from the five glomerulonephritis patients appeared as numerous and voluminous electron‐dense deposits in the mesangium or in its vicinity, just underneath the glomerular basal membrane (Figure 3a). In the five FSGS patients small, electron‐dense immune deposits were seen in the mesangium (Figure 3c), at times associated with intramembranous electron‐dense hyaline material (Figure 3d). Neither the immunoglobulin deposits nor the hyaline material from the patients with renal disease exhibited a granular pattern or the halo. The halo was not detected even during resorption of the immune deposits (Figure 3b).

The number of GOM deposits surrounded by the halo did not correlate with the number of deposits in the sample; in addition, there was no correlation between halo thickness and deposit size. The GOM deposits were either homogeneously osmiophilic or they had a mixed morphology. The uniformly dense deposits tended to be more osmiophilic if they were located close to the cell membrane (Figure 1b,c), and less osmiophilic if they lay farther away from it (Figure 1d). In the deposits characterized by heterogeneous osmiophilia, the side facing the infolding was more electron‐dense and exhibited a smooth outline, whereas the outer side was less osmiophilic, looser, and showed a more irregular profile (Figures 1a and 2d). However, the degree of osmiophilia did not seem to correlate with the presence/absence of the halo.

## 4 Discussion

This ultrastructural study of tissue biopsies from 13 CADASIL patients confirms the presence and provides a detailed description of an electron‐lucent halo surrounding several GOM deposits in the arteries of skin, muscle, kidney, and pericardium. An electron‐lucent space had previously been described between the GOM and the cell membrane in skeletal muscle (Ruchoux et al., 1995) and skin (Ishiko et al., 2005) from CADASIL patients. However, in this study, the halo was also detected on the outer side in all biopsy types examined; moreover, some deposits were completely surrounded, a finding that may be related to the plane of section. Recently, our group has identified GOM‐containing pseudoinclusions in 70% of our 13 CADASIL patients (Morroni, Lorenzi, Castellucci, Ragno, & Scarpelli, 2014), where also these GOM deposits were often surrounded by an electron‐lucent halo. Comparison of the glomerular GOM deposits of the CADASIL patients with the mesangial immunoglobulin deposits and the paramesangial hyaline deposits found in the kidneys of patients with glomerulonephritis or FSGS provided further confirmation of the association of the halo with GOM. Despite the identical tissue type, similar sample preservation, and simultaneous analysis, only GOM from the former patients showed an electron‐lucent halo. In addition, the extracellular deposits produced by a protein aggregation process similar to GOM formation, that is, the amyloid beta (Aß) plaques documented in neurons of patients with Alzheimer's disease, lack the halo (Yamaguchi, Nakazato, Hirai, Shoji, & Harigaya, 1989). This further demonstrates that the halo around GOM is not an artifact, since its presence is not necessarily connected to a particular site or type of aggregation.

Another notable ultrastructural feature of the GOM deposits, both those located in muscle cell infoldings and those scattered in the interstitium, is that there were two types: uniformly electron‐dense and mixed ones. GOM deposits with inhomogeneous morphology have also recently been described by Lewandowska et al. (2011). Varying osmiophilia depending on site—greater in the deposits closer to the cell membrane and reduced in those scattered in the interstitium—has also been described (Ishiko et al., 2005; Ruchoux et al., 1995).

Analysis of the present data suggests that the halo around GOM deposits might isolate them and thus interfere both with ubiquitination and transendocytosis of mutant NOTCH3 (Dziewulska & Rafalowska, 2008; Watanabe‐Hosomi, Watanabe, Tanaka, Nakagawa, & Mizuno, 2012), which are key steps for physiological GOM clearance, pointing at a possible, crucial role for the halo in CADASIL pathogenesis. In this framework, the nonuniform osmiophilia of GOM deposits could be interpreted as defective or incomplete clearance causing formation of partially resorbed GOM deposits.

Ruchoux et al. (1995) interpreted the halo they described (and designated lucida lamina) as remaining VSMC processes and cytoplasm. In view of the considerations of Monet‐Leprêtre et al. (2013) about the accumulation of tissue inhibitor of metalloproteinase 3 (TIMP3) on the perimeter of GOM deposits, it is conceivable that a more densely packed extracellular matrix around the GOM could be the result of reduced metalloproteinase 3 activity. The halo could represent the result of this event.

In conclusion, the present findings confirm some previously reported morphological features of GOM, in particular, the evidence of inhomogeneous electron density. In addition, they confirm the existence and provide a detailed description of an electron‐lucent halo around several GOM deposits. The halo might be the morphological evidence and the cause of an aberrant NOTCH3 processing, which has already been hypothesized in CADASIL (Chabriat, Joutel, Dichgans, Tournier‐Lasserve, & Bousser, 2009; Louvi, Arboleda‐Velasquez, & Artavanis‐Tsakonas, 2006). We believe that the halo surrounding the GOM may be an important ultrastructural marker, which can be useful when GOM deposits are not easily recognized. In this connection, slightly electron‐dense deposits consistent with GOM have recently been described in VSMC indentations in a skin biopsy from an elderly CADASIL patient with anxiety disorder: it was the electron‐lucent halo around the deposits that helped their identification as GOM (Ragno et al., 2016). This suggests that reevaluation of some CADASIL cases, where GOM deposits have not been identified (Erro et al., 2015; Moccia et al., 2015), could in fact lead to discovery of the halo, hence of the deposits. Thus, we do not agree with Erro et al. (2015) that GOM deposits may be an epiphenomenon of CADASIL disease.

Since GOM clearance would be a key therapeutic strategy for CADASIL, especially given the toxic action and key role of GOM in altering perivascular drainage (Carare, Hawkes, Jeffrey, Kalaria, & Weller, 2013), further research—particularly molecular studies—is needed to investigate the nature of the halo and its functional role.

## Conflict of Interest

All the authors declare that they have no conflict of interest.

## Author Contributions

TL: Analysis and interpretation of the data; drafting the manuscript. MR: Analysis and interpretation of the data; revising the manuscript for intellectual content. FP, CC: Analysis and interpretation of the data. MS: Revising the manuscript for intellectual content. MM: Design and conceptualization of the study; analysis and interpretation of the data; drafting the manuscript.
